# Stepping Up: Predictors of ‘Stepping’ within an iCBT Stepped-Care Intervention for Depression

**DOI:** 10.3390/ijerph16234689

**Published:** 2019-11-25

**Authors:** Jennifer Nicholas, Kathryn E. Ringland, Andrea K. Graham, Ashley A. Knapp, Emily G. Lattie, Mary J. Kwasny, David C. Mohr

**Affiliations:** 1 Center for Behavioral Intervention Technologies, Department of Preventive Medicine, Northwestern University, Chicago, IL 60611, USA; kathrynringland@northwestern.edu (K.E.R.); andrea.graham@northwestern.edu (A.K.G.); ashley.knapp@northwestern.edu (A.A.K.); emily.lattie@northwestern.edu (E.G.L.); d-mohr@northwestern.edu (D.C.M.); 2 Department of Communication Studies, Northwestern University, Chicago, IL 60611, USA; 3 Department of Medical Social Sciences, Northwestern University, Chicago, IL 60611, USA; 4 Biostatistics Collaboration Center, Preventive Medicine, Feinberg School of Medicine, Northwestern University, Chicago, IL 60611, USA; m-kwasny@northwestern.edu

**Keywords:** stepped-care, depression, digital mental health, eHealth, telepsychiatry

## Abstract

Internet-based cognitive behavioral therapy (iCBT) may overcome barriers to mental health care and has proven efficacious. However, this approach currently exists outside the existing mental health care delivery system. Stepped care is a proposed framework for integrating digital mental health (DMH) into health systems by initiating iCBT and “stepping up” care to a more intensive intervention should iCBT prove ineffective. This study explores pre-treatment factors associated with reaching stepping criteria among patients receiving iCBT. This exploratory analysis of a stepped care arm of a larger randomized trial examined participants who were stepped to a more intensive intervention if they did not respond to iCBT. The association of pre-treatment factors on stepping were examined using Kruskal–Wallis and Chi-square tests. One-fifth of the 151 participants met criteria for stepping within the 20-week treatment period. Only pre-treatment depression severity and treatment preference were associated with increased likelihood of stepping (*p* = 0.049 and 0.048, respectively). The low number of individuals who stepped provides support for iCBT as an effective, low intensity treatment for depression. The modest association of pre-treatment depression and preference to not receive iCBT may be useful in identifying patients who are less likely to respond.

## 1. Introduction

Psychotherapy is now the front line, gold standard treatment of common mental health disorders, such as depression and anxiety, within evidence-based medicine [1]. Yet, due to barriers including access, cost, and stigma, estimates suggest that up to two-thirds of individuals with depression or anxiety do not access or receive evidence-based care such as cognitive behavioral therapy (CBT; [2]). In an effort to increase access to, and fidelity of, evidence-based treatment, alternatives to traditional face-to-face models of care have been sought. With the increase in the access and availability of the internet over the last two decades, internet-based interventions delivering CBT (iCBT) have emerged [3]. Primarily derived from face-to-face CBT protocols, iCBT interventions are module-based CBT programs that progress users through psychoeducation about the cognitive-behavioral model of depression and teach core cognitive and behavioral skills, often via interactive exercises (e.g., identifying and challenging negative thoughts) [4]. In addition, iCBT interventions may also integrate guidance from clinicians or peer workers, with guided iCBT reporting better engagement than unguided CBT [5]. Regardless of the presence or absence of guidance, iCBT now has an extensive evidence-base, showing efficacy similar to face-to-face CBT at reducing symptoms of a range of mental health conditions, in particular, depression and anxiety [6]. Although efficacious, iCBT was developed outside the healthcare system, and consequently, most use occurs within direct to consumer models. With the success of iCBT, attention has turned to how such interventions can be integrated within the larger mental health system of care.

One possible solution is stepped care. Stepped care models feature a hierarchy of interventions or types of care that individuals move between based on assessed need. Stepped care models are fundamentally based on two foundations. First, that individuals receive the “least restrictive” treatment that is expected to be effective [7]. Within the context of mental health care, least restrictive has come to mean low-intensity treatments that require less time from mental health professionals [8]. Second, critical to the success of stepped care, is that people receive the appropriate level of intervention. Therefore, the second foundation of stepped care is that of ongoing assessment [7]. Individuals who do not respond to their current level of treatment are ‘stepped up’ to more intensive intervention. Internet-based interventions, such as iCBT, fit well within stepped care frameworks as they accommodate these two foundations. ICBT programs are “least restrictive”; they are generally conceived as low-intensity interventions, administered with no, or minimal, clinical guidance and are broadly accessible. Further, able to present and prompt users to complete assessments, iCBT programs also facilitate ongoing assessment.

Within mental health, there have been two approaches to the implementation of stepped care into health systems: stratified and progressive. Stratified stepped care models assess an individual’s needs and then assign them to the least intense intervention within the model that meets those needs. In contrast, progressive stepped care initially offers the lowest intensity intervention to all individuals and steps those who do not respond to treatment. The stratified approach may ultimately provide a more efficient use of resources, but requires knowing which patient profiles would benefit from particular interventions before the interventions are assigned. Within the mental health literature, inconsistency exists regarding for whom low-intensity interventions, such as iCBT, are considered most effective. Generally, iCBT is targeted towards individuals with mild-to-moderate symptoms [9,10]. However, a meta-analysis of patient data from low-intensity interventions for depression, including remotely delivered interventions such as iCBT, found individuals with more severe pre-treatment depression experience similar benefit from low-intensity interventions including iCBT as those with mild-moderate symptoms [11].

Our recent study evaluated a progressive stepped care intervention for depression, in which participant-initiated treatment with iCBT and were stepped to a telephone-based CBT intervention (tCBT) if they did not meet criteria for improvement [12]. Results indicated that stepped care was non-inferior to tCBT for treating depression, but cost approximately as much [12]. However, due to the inconsistency in the literature regarding the usefulness of iCBT for more severe symptoms, our main outcomes do not elucidate whether some participants would have been better served under a stratified approach, in which participants with more severe symptoms initiate treatment with tCBT rather than iCBT. In order to meaningfully assist those with mental health disorders and make best use of available treatment options, understanding the pre-treatment factors that are associated with stepping up within stepped care is critical. The purpose of this study was to explore the pre-treatment factors associated with meeting step criteria within a progressive stepped care intervention with iCBT as the first stage. 

## 2. Materials and Methods

### 2.1. Participants and Procedure

This study is a secondary analysis of a randomized trial comparing a stepped care intervention, with iCBT as the first step and tCBT as the second step, to tCBT [12]. This study examined participants of the stepped care intervention arm only. A detailed description of the study methods has been published [12]. In brief, participants were recruited between February 2015 and May 2017 via public advertisements, connections within healthcare systems in the USA, and social media campaigns. Individuals who were over 18 years of age, met criteria for major depressive episode on the Mini International Neuropsychiatric Interview (MINI; [13]), scored greater than 12 on the Quick Inventory of Depression Symptomatology—Clinician Rated (QIDS; [14]), could read and speak English, had access to a computer, and no diagnosis of severe mental health disorders, current suicidal ideation, or medication changes or psychotherapy were eligible to participate. All procedures were approved by the Northwestern University Institutional Review Board, and all participants provided online informed consent. The trial was monitored by an independent Data Safety Monitoring Board. This study was approved by the Institutional Review Board at Northwestern University (ethical approval number: STU00064411).

### 2.2. Intervention

Stepped care treatment was delivered for up to 20 weeks. Within the stepped care intervention, all participants received an iCBT intervention called ThinkFeelDo, known to be efficacious in reducing symptoms of depression and anxiety [15,16,17]. ThinkFeelDo is a modular CBT-based online program designed to provide psychoeducation and facilitate skill building through interactive tools. Content is delivered via animated audio-visual clips and short text information. In addition, participants received weekly coaching with a graduate-level therapist who communicated with participants via telephone and through the online platform to encourage engagement and facilitate skill development. Coaching was based on a manualized supportive accountability model [18], with calls lasting approximately 10–15 min.

Participants who did not experience improvement in symptoms were ‘stepped up’ to a more intensive intervention (tCBT). Within this trial, participants were offered to step up if they met criteria that predicted non-response [19], defined as a score on the 9-item Patient Health Questionnaire (PHQ-9; [20]: (i) >16 for two consecutive weeks from weeks 4–8, (ii) >12 for two consecutive weeks from weeks 9–13, or (iii) >8 for two consecutive weeks after week 13. Participants were not stepped after week 16. Participants who met criteria for stepping and agreed to be stepped began the tCBT intervention with their iCBT coach as their therapist. All participants that met criteria agreed to be stepped. The tCBT intervention was based on a manualized protocol with demonstrated non-inferiority to face-to-face psychotherapy. Therapy was provided in a single hour-long session each week over the phone accompanied by a workbook, and covered cognitive-behavioral strategies focused on reframing unhelpful automatic thoughts, pacing activities, time management skills, planning and organization, increasing pleasant events, anxiety management, anger management, and social skills training.

### 2.3. Measures

Participant demographics considered in this study were age and sex. Depression was measured using the PHQ-9, a self-report measure for depression symptom severity with demonstrated reliability and validity [20]. Comorbid anxiety symptom severity was measured via self-report using the Generalized Anxiety Disorder (GAD)-7 [21]. Current pharmacological and recent psychological treatment (within the last 3 months) were assessed. Specifically, use of current pharmacological treatment was assessed with the self-report question, “are you currently taking any antidepressant medications?” Recent psychological treatment was assessed using an item from the Cornell Service Use Index [22], “which of the following Psychological Services have you used in the past 3 months?” Participants perceived ability to cope with stress was measured pre-treatment using the Coping Self-Efficacy Scale [23]. Overall physical health and wellness was measured using the Veterans Rand-36 Item Health Survey (VR-36; [24]). Component scores assessing physical functioning (physical component score, PCS) and mental health (mental component score, MCS) were used rather than the total score. Before commencing treatment, participants were asked which treatment they would prefer, iCBT, tCBT, or no preference. As an indication of comfort using technology, participants were asked “how often do you access the internet using your laptop or desktop?” and responded on a Likert scale ranging from 1–5, with 1 being less than once per week, and 5 several times per day. To assess stepping, participants who met criteria to transfer from iCBT–tCBT at any point throughout the 20-week intervention period were considered ‘steppers’. Individuals who stepped before week ten were grouped as ‘early steppers’, while those who stepped during the remainder of the intervention were designated ‘late steppers’. Participants that remained in iCBT for the whole intervention were ‘non-steppers’, and either completed 20 weeks of iCBT, or met criteria for remission (PHQ-9 < 5 for two consecutive weeks) and discontinued therapy.

### 2.4. Data Analysis

Analysis included all participants who initiated iCBT. Treatment preference, measured as a preference for iCBT, tCBT, or no preference, was recoded into a dichotomous variable for analysis. Those who indicated a preference for tCBT (i.e., those participants who initially received a treatment counter to their preference) were kept separate, and those who preferred iCBT or had no preference (i.e., either those who initially received their preferred treatment, or those with no inclination for either treatment) were combined, thereby focusing the analysis on those who did not receive their preferred treatment. Similarly, due to the high frequency of internet access using computer in the sample, frequency of use was dichotomised by combining those who accessed the internet via their computer once a day or less (responses 1–4) and separate from those who accessed several times a day (response 5). As an indication of treatment status, current pharmacological treatment and psychological treatment within the last 3 months were combined for analysis, to indicate participants who were either currently receiving or had a recent history of treatment (either psychological or pharmacological) for depression. Due to the small sample size within the stepping groups, (early steppers, later steppers, non-steppers), Kruskal–Wallis test for independence was used to investigate differences between the stepping groups on continuous pre-treatment variables. Kruskal–Wallis is the non-parametric alternative to a one-way ANOVA that compares the mean rank for each group rather than the mean. Chi-square, a common test used to explore the relationship between two categorical variables, was used to determine differences between stepping groups related to gender, treatment preference, and recent treatment history, and differences between treatment preference and frequency of computer use. A Mann-Whitney U test, a non-parametric alternative to the *t*-test for independent samples that compares medians rather than means, was used to explore differences between treatment preference on pre-treatment depression severity. Analyses were conducted in SPSS version 25 (IBM Armonk, NY 10504, USA). Alpha was set to *p* < 0.05.

## 3. Results

### 3.1. Participants

Of the 312 randomized participants in the trial, 151 participated in the iCBT stepped care arm of the study. Characteristics of all randomized participants and study flow have been published [12]. The majority of iCBT stepped care participants were female (74.8%), identified as White (90.1%), were employed (65.6%), and had a median (IQR) age of 33 years (27–49). Most participants were college educated (72.8%). Median (IQR) pre-treatment PHQ-9 and GAD-7 scores were 16 (14–19) and 13 (9–16), respectively. At pre-treatment, the majority of participants were not taking any antidepressant medication (57.0%), had not participated in psychotherapy in the last 3 months (89.4%), and approximately half (51%) were not currently and had not recently received treatment of either antidepressants or psychotherapy.

In selecting a treatment preference, 50 participants (33.1%) preferred tCBT, 48 (31.8%) iCBT, and 53 (35.1%) had no preference. Treatment preference was not associated with pre-treatment depression severity, with no significant difference in depression severity between those who received their preferred treatment (16 (14–19), *n* = 101) and those who did not (i.e., preferred tCBT; 16.5 (14–20.25), *n* = 50) U = 2287, z = −0.95, *p* = 0.35. Across the sample, computer use was high with 25 (75.8%) of the participants who preferred tCBT, 29 (82.9%) of the participants who preferred iCBT, and 29 (78.4%) of the participants who had no preference indicating that they used their computer to access the internet several times per day. Frequency of computer use, however, was not significantly associated with treatment preference (χ^2^ (2, *n* = 105) = 0.53, *p* = 0.76, phi = −0.07) or pre-treatment depression (U = 909.5, z = −0.028, *p* = 0.97).

### 3.2. Stepping during Treatment

During the intervention, the majority of participants (76.2%) did not meet criteria to be stepped up from iCBT. The remaining 23.8% of participants (*n* = 36) stepped during the treatment period, 18 (11.9%) before the halfway mark of 10 weeks (early steppers), and 18 (11.9%) after the tenth week (late steppers). Median depression scores over the course of the intervention show that early and late steppers take longer to show pronounced response to treatment (Table 1).

### 3.3. Pre-treatment Factors Associated with Stepping

Pre-treatment PHQ-9 score was significantly associated with meeting stepping criteria within the treatment period (χ^2^ (2, *n* = 151) = 6.04, *p* = 0.049). Participants who stepped early were more likely to have a higher median (IQR) pre-treatment depression severity, than those who stepped late or did not step (Table 1). Pre-randomization preference for tCBT treatment was also significantly associated with stepping (χ^2^ (2, *n* = 151) = 6.09, *p* = 0.048). Individuals who preferred tCBT, and therefore did not initially receive their preferred treatment, were more likely to meet criteria for stepping (36% stepped) than those who indicated a preference for iCBT or had no preference (17.8% stepped). No other pre-treatment factors were significantly associated with stepping (all *p*’s > 0.246).

## 4. Discussion

How best to implement DMH interventions into traditional care settings is a major consideration for reducing the prevalence of mental health problems. Given the benefits of stepped-care approaches for appropriately tailoring resources to individuals’ needs and conserving resources, we sought to examine pre-treatment factors which may predict a lack of response to low-intensity intervention and therefore a need for stepping, elucidating factors that may guide a stratified stepped care approach. However, our results indicate that this is a complicated problem. Foremost, only a fifth of our participants met criteria to step during the study, indicating that iCBT was effective for the vast majority of individuals. Among those that did step, there was a modest effect of pre-treatment depression severity, whereby those who had more severe pre-treatment symptoms were more likely to step. However, this effect may only hold for those who stepped early. Those who stepped were also more likely to have indicated a preference for tCBT and therefore participated in a non-preferred intervention.

Given the modest effect of pre-treatment depression severity on stepping, and in light of the limited number of individuals that met the criteria to step, our results require replication in a larger sample before any clinical determination regarding progressive or stratified implementations of stepped care can be made. If these results are confirmed, we believe there would be no clear recommendation that iCBT programs should not act as a first line intervention in the treatment of depression. Whilst resource availability within the integrated care system would also likely affect this recommendation, services are over-stretched in most care settings. Therefore, a progressive model with iCBT as a first step may be the most feasible approach to DMH support within health contexts, as supported by previous results of the broad efficacy of iCBT interventions [11]. However, if clinicians are available to deliver more intensive (e.g., in-person) treatment, then individuals with severe depression could feasibly skip the lowest intensity intervention and begin with more intensive clinical care.

Interestingly, treatment preference influenced individuals’ likelihood of meeting step criteria during the intervention. Importantly, this association could not be explained by symptom severity; individuals with more severe depression were no more likely to express a preference for tCBT as those with milder depression. While it may be that participants had a strong preference for tCBT, it is also possible that some participants had a preconceived idea that an internet-based, minimally guided tool could not impact their symptoms or meet their needs. Research from technology fields, such as human-computer interaction, highlights the importance of users’ frame of reference when they encounter new technologies [25]. However, little attention has been paid to framing within DMH, particularly surrounding the introduction of these tools to individuals living with mental health conditions. To the extent that preference for tCBT results from suspicion or misunderstanding about iCBT, education around DMH interventions could mitigate this effect.

Limitations of this study also require mentioning. Foremost, the small number of participants who met criteria to step reduced our ability to detect and interpret predictors of stepping within a stepped care intervention. Although these findings provide continued support for iCBT as a first-line treatment approach [11], results require replication before recommendations of implementation models can be made. Second, our participants were compensated for study participation (i.e., completion of research assessments, but for not treatment engagement); therefore, engagement with, and potentially benefit of, the intervention may have been enhanced compared to how people engage with and are incentivized to continue care in real world settings. In a further threat to generalisability, our participants predominantly identified as white, female, were employed, educated, and likely highly motivated, due to the high degree of self-selection into the study. However, we believe the high degree of self-selection supports the importance of a mental health landscape with a range of service delivery options that cater to individual preference, in the hope that all individuals have an avenue to care regardless of real or perceived barriers. The high proportion of female participants is common within studies of online interventions [6,26], potentially due to the higher incidence of depression and a greater willingness to seek help in this group. In light of these limitations, future research should consider the predictors of stepping with larger, more diverse samples, ideally within stepped care interventions integrated in health care systems where the intervention would likely be implemented in the future. Future work would also benefit from integrating a qualitative component. Qualitative research could elucidate user perspectives of these still nascent methods of service delivery (iCBT and tCBT) and models of care (stepped care). Further, qualitative work exploring participant experiences of iCBT and tCBT would also provide valuable insight regarding the use of these treatments in care settings that could guide the implementation and tailoring of these interventions.

## 5. Conclusions

Our results indicate that although higher pre-treatment depression predicted stepping, the effect was small and the majority of participants did not need to step from iCBT to the higher intensity intervention. While participants who preferred tCBT were more likely to step, it is unclear if this was a strong desire for tCBT or lack of confidence in iCBT, which would be a potentially malleable factor that could increase the efficacy of iCBT. This would suggest greater attention be paid to how people currently view digital mental health programs and how these perspectives potentially affect people’s engagement, experience, and response to such programs. Although the current study enhances our understanding of how we can use stepped care models for DMH integration in clinical settings, ultimately, larger studies are required to confirm and extend these findings.

## Figures and Tables

**Table 1:** Median (IQR) Depression Scores for Each Group Across the Intervention Period.

Time Point	Non-Steppers	Early Steppers	Late Steppers
Start of treatment	16 (13.5–19)	19 (14.5–22.25)	16 (16–18.25)
Week 4	8.5 (5–12)	17.5 (17–19)	13 (11–16)
Week 9	8 (5–10)	14.5 (12–17.5)	12 (7–15)
Week 13	8 (5–10)	10 (7–19)	11 (7–14)
End of treatment (week 20)	6 (4–9)	9 (7–18)	11 (6.25–14.75)
